# Urban commuting dynamics in response to public transit upgrades: A big data approach

**DOI:** 10.1371/journal.pone.0223650

**Published:** 2019-10-17

**Authors:** Qi-Li Gao, Qing-Quan Li, Yan Zhuang, Yang Yue, Zhen-Zhen Liu, Shui-Quan Li, Daniel Sui

**Affiliations:** 1 State Key Laboratory of Information Engineering in Surveying, Mapping and Remote Sensing, Wuhan University, Wuhan, P.R. China; 2 Shenzhen Key Laboratory of Spatial Smart Sensing and Services, Department of Urban Informatics, School of Architecture and Urban Planning, Shenzhen University, Shenzhen, P.R. China; 3 Guangdong Key Laboratory of Urban Informatics, Shenzhen University, Shenzhen, P.R. China; 4 College of Computer Science and Software Engineering, Shenzhen University, Shenzhen, P.R. China; 5 Department of Geosciences, University of Arkansas, Fayetteville, AR, United States of America; Johns Hopkins University, UNITED STATES

## Abstract

Public transit, especially urban rail systems, plays a vital role in shaping commuting patterns. Compared with census data and survey data, large-scale and real-time big data can track the impacts of urban policy implementations at finer spatial and temporal scales. Therefore, this study proposed a multi-level analytical framework using transit smartcard data to examine urban commuting dynamics in response to rail transit upgrades. The study area was Shenzhen, one of the most highly urbanized and densely populated cities in China, which provides the opportunity to examine the effects of rail transit upgrades on commuting patterns in a rapidly developing urban context. Changes in commuting patterns were examined at three levels: city, region, and individual. At the city level, we considered the average commuting time, commuting speed, and commuting distance across the whole city. At the region level, we analyzed changes in the job accessibility of residential zones. Finally, this study evaluated the potential effects of rail transit upgrades on the jobs-housing relationship at the individual level. Difference-in-difference models were used for causal inference between rail transit upgrades and commuting patterns. In the very short term, the opening of new rail transit lines resulted in no significant changes in overall commuting patterns across the whole city; however, two effects of rail transit upgrades on commuting patterns were identified. First, rail transit upgrades enhanced regional connectivity between residential zones and employment centers, thus improving job accessibility. Second, rail transit improvement increased the commuting distances of individuals and contributed to the separation of workplaces and residences. This study provides meaningful insights into the effects of rail transit upgrades on commuting patterns.

## Introduction

Public transit systems play significant roles in urban development and in shaping commuting behaviors [1, 2]. In high-density populated cities such as New York, Paris, Tokyo, and Hong Kong, public transit is the travel mode most frequently used for daily commuting. For instance, more than half of the people in New York use the public transportation system [3]; in Tokyo, more than 70% of the population travels on public transit [4], and in Hong Kong, over 90% of people use transit [5]. In particular, the high capacity and efficiency of rail transit (e.g. metro and light rail) has led it to enjoy priority in the transportation and land use development strategies of high-density or compact cities [6, 7]. Worldwide, many cities have made massive investments in improving their urban rail transit systems, especially in fast-growing developing countries [8–10].

It is widely believed that efficient public transit can improve job accessibility and address mobility problems [11, 12]. A growing quantity of literature has investigated the connection between rail system improvement and commuting behavior, particularly changes in public transit usage [2, 13]. In the U.S. context, transit ridership has been declining rapidly despite significant improvements in public infrastructure [8]. However, cities like Paris, London, and Tokyo have highly efficient urban rail transit systems that provide effective connections between city center and outer suburbs [14]. Public transit improvements also affect commuting patterns by changing residence and jobs locations [15]. Specifically, improvements in urban transportation infrastructure can affect the physical layout of cities by changing the distributions of residences and employment opportunities. Some evidence indicates that expansion of a transport network contributes to decentralization of both the urban population and employment, which produces changes in commuting patterns [16].

In recent decades, China has witnessed rapid urban growth and construction of transportation infrastructure. This has resulted in great changes in the spatial organization of residences and employment opportunities, leading to changes in commuting patterns [17–19]. Unlike in Western cities, a large proportion of low-income residents in Chinese cities live in the suburban areas and outskirts, and employment decentralization is not as pronounced. Most job opportunities remain concentrated in the inner city, especially for low-skilled workers [20]. As a result, significant commuting inequality appeared in large Chinese cities, and many obstacles prevent people from moving closer to employment centers, particularly limited affordable housing resources in the city center [21–23]. As a result, large Chinese cities have developed significant commuting inequality. In response to these issues, local governments have been actively building rail transit systems, endeavoring to strengthen the geographical connection of suburban residents with employment centers in the inner city [9]. These projects provide opportunities to examine the effects of rail transit upgrades on commuting patterns.

Surveys and censuses are two data sources commonly used in traditional commuting studies. However, individual demographics and survey data are usually difficult to access, and sample sizes are always limited. Additionally, it is impossible to estimate effects on individual commuting behavior from aggregated census data; thus, census data is usually used to examine changing commuting patterns at either national or regional levels. In recent years, the development of information and communication technology has enabled the continuous generation of various large-sample and fine-scale movement datasets, which can effectively capture human behaviors in daily life [24]. One such source for urban big data is public transit smartcard data, which records users’ travel behaviors. Personal commuting information (e.g. residence, workplace and commuting time) can be extracted according to daily travel regularity [25, 26]. More importantly, smartcard records are automatically generated when travel occurs, making it possible to examine commuting dynamics and the jobs-housing relationship at finer temporal resolutions than is feasible with slowly upgraded conventional data. In this sense, real-time movement data enable us to evaluate the short-term and long-term impacts of urban policy implementations in an effective way.

This study aims to supply evidence regarding the influence of new rail transit lines on commuting patterns through a case study of Shenzhen, China. Due to the interaction of various factors influencing urban commuting dynamics, the effects of rail transit cannot be intuitively reflected in the overall commuting patterns. Hence, this study introduces a multi-level and individual-based analytical framework to explore commuting dynamics from different perspectives. The proposed methodology can examine how and to what extent rail transit upgrades affect urban commuting patterns. Specifically, this study aims to address issues at three levels. 1) The city level: what was the average commuting time, commuting speed, and commuting distance across the whole city before and after rail transit upgrades? 2) The region level: what was the change in accessibility of residential zones to employment centers with rail transit upgrades? 3) The individual level: how did the newly opened rail transit lines reshape individual jobs-housing relationships? Difference-in-difference models were used to explore the associations between rail transit upgrades and manifestations of commuting behaviors. This study contributes to existing research in two ways. Firstly, this study proposes an analytical framework for using big data to identify commuting dynamics in response to urban rail transit upgrades. Secondly, the findings in this study illustrate how improvements in rail transit reshape urban commuting patterns in a rapidly- developing country and provide some evidence for urban policies and planning strategies.

### Study area

**Fig 1:**
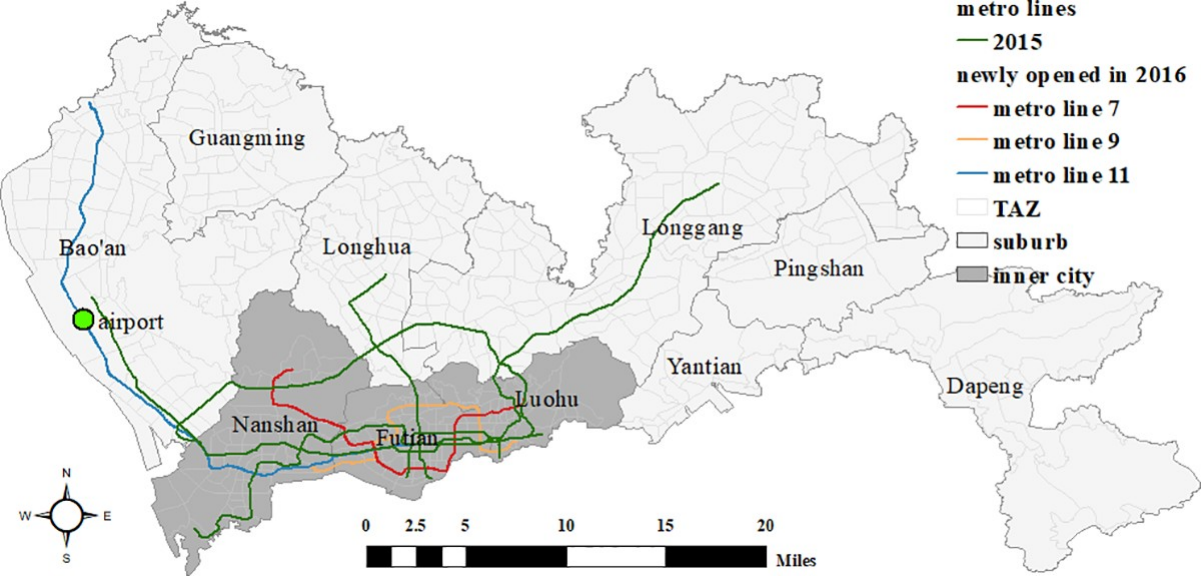
Study area and metro lines.

The study area, Shenzhen, covers 1997.47 km^2^, consisting of ten districts and 491 traffic analysis zones (TAZ) (**Fig 1**). As one of the most highly urbanized and densely populated cities in China, Shenzhen housed 11.4 million permanent residents in 2015, which increased to 11.9 million the following year (Shenzhen Statistical Yearbook 2016, 2017). The city center is constituted of three central districts, Nanshan, Futian and Luohu. Due to urban sprawl, the city’s public transit systems are highly developed. While Shenzhen’s bus system and road networks are relatively stable, its rail transit system has expanded rapidly. At present, it has eight rail transit lines, covering a total of 285 kilometers. Five of the eight metro lines were opened before 2015; the other three (i.e. metro lines 7, 9, and 11) were opened in 2016, increasing the number of metro stations from 131 to 199. Specifically, metro lines 7 and 9 opened on October 28 and mainly serve the central areas. Metro line 11, opened on June 28 and also known as the “airport line”, connects the inner city and suburbs. In addition, more than twenty additional metro lines are under construction or being planned for construction in the coming ten years; this is due to the ‘70/70’ transport plan that calls for 70% of daily travel to be made by public transit, and for 70% of those trips to be made by rail transit. This rapid and ongoing development of rail infrastructure may continuously change the commuting patterns and spatial layout of residences and employment opportunities in Shenzhen over the next ten years, which provides us with excellent opportunities to examine how rail transit upgrades affect urban commuting dynamics in a high-density urban context.

### Data and methodology

Public transit smartcard data from two periods were used to explore dynamic changes in the commuting patterns and jobs-housing relationships of public transit commuters. The first period is 5–11 Jan. 2015, before the opening of three new metro lines (i.e. metro lines 7, 9 and 11), and the second period is 22–28 Nov. 2016, after their opening. When a rider uses a smartcard to pay for a public transit trip, the unique card number, boarding time, boarding vehicle number (bus trip), and boarding/get-off station (metro trip) are automatically recorded. For bus trips, the boarding station and get-off station were inferred from bus GPS data and bus line data. For multimodal transfers, bus trips and metro trips were merged based on the unique card numbers.

**Fig 2:**
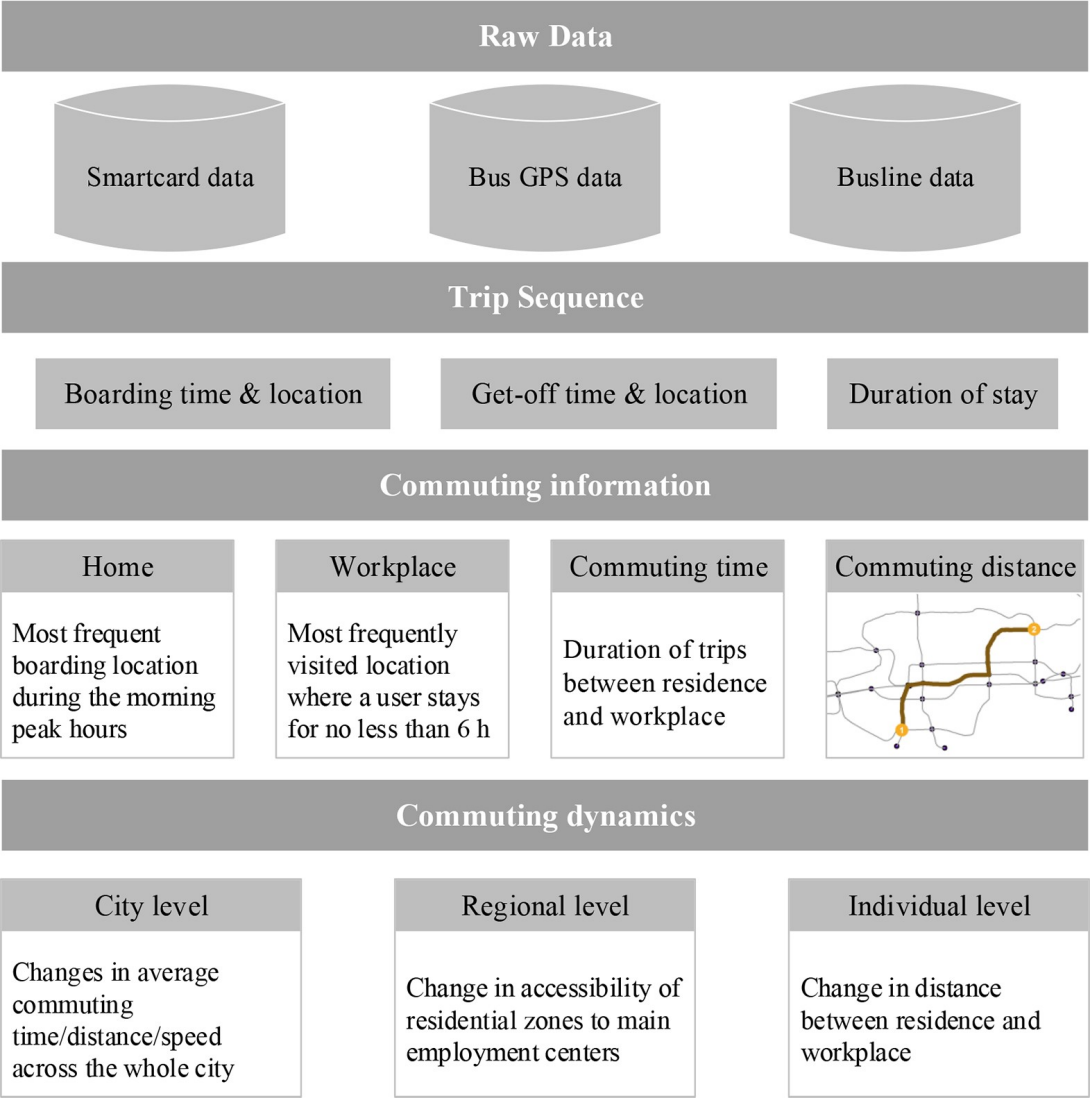
Analytical framework of this study.

**Fig 2** shows the analytical framework of this study. Typical public transit riders often commute between relatively stable places (i.e. residence and workplace) at relatively fixed times; therefore, residences and workplaces can be identified based on specific commuting regularity and spatial-temporal repeat patterns. This study defines as residence the most frequent boarding station (visited at least three times in a week) of a smartcard user during morning peak hours (6:00~10:00 am). Similarly, a rider’s workplace is defined as the most frequently visited place where the rider stayed for six hours or more. Commuting time is the duration of trips between one’s residence and workplace. For a bus rider, the travel distance is defined as the actual network distance based on bus lines, and for a subway rider as the length of the shortest path based on metro lines.

According to the above definitions, approximately 0.63 million commuters were identified out of a total ridership of 2.48 million during the first period, and over 0.96 million were identified from a ridership of 2.57 million during the second period. Given that our analysis is based on comparisons between two identified groups over the two periods, the two groups should have similar demographics. Evidence shows that 2% sampling rate is sufficient in modeling intra-city human mobility patterns (e.g., travel distance and travel time) using public transit smart card data [27]. Therefore, we consider the identified two groups with more than 20% sampling rates are demographically and statistically similar in this study.

Urban commuting dynamics are jointly determined by co-location of residences and jobs and transport systems. The development of rail transit affects both commuting efficiency and individual jobs-housing locations. However, individual relocations are also influenced by other factors, such housing factors and individual socioeconomic levels. Due to the interactions of various influencing factors, the effects of rail transit on the commuting dynamics cannot be intuitively reflected in the overall patterns. Based on this concern, this study designs a multi-level and individual-based analytical framework to explore commuting dynamics to examine how and to what extent rail transit upgrades affect urban commuting patterns from different perspectives. At the city level, we focus on overall commuting patterns across the whole city, including average commuting time, commuting distance, and commuting speed. At the region level, we concentrate on the changes in commuting efficiency and job accessibility of residential zones. We further detect which areas enjoyed prominent reductions in commuting time. At the individual level, we use commuting distance as a proxy for jobs-housing separation to explore how rail transit upgrades have influenced the spatial relationships of individuals’ residences and workplaces.

### Analysis and findings

#### Overall commuting dynamics

**Fig 3:**
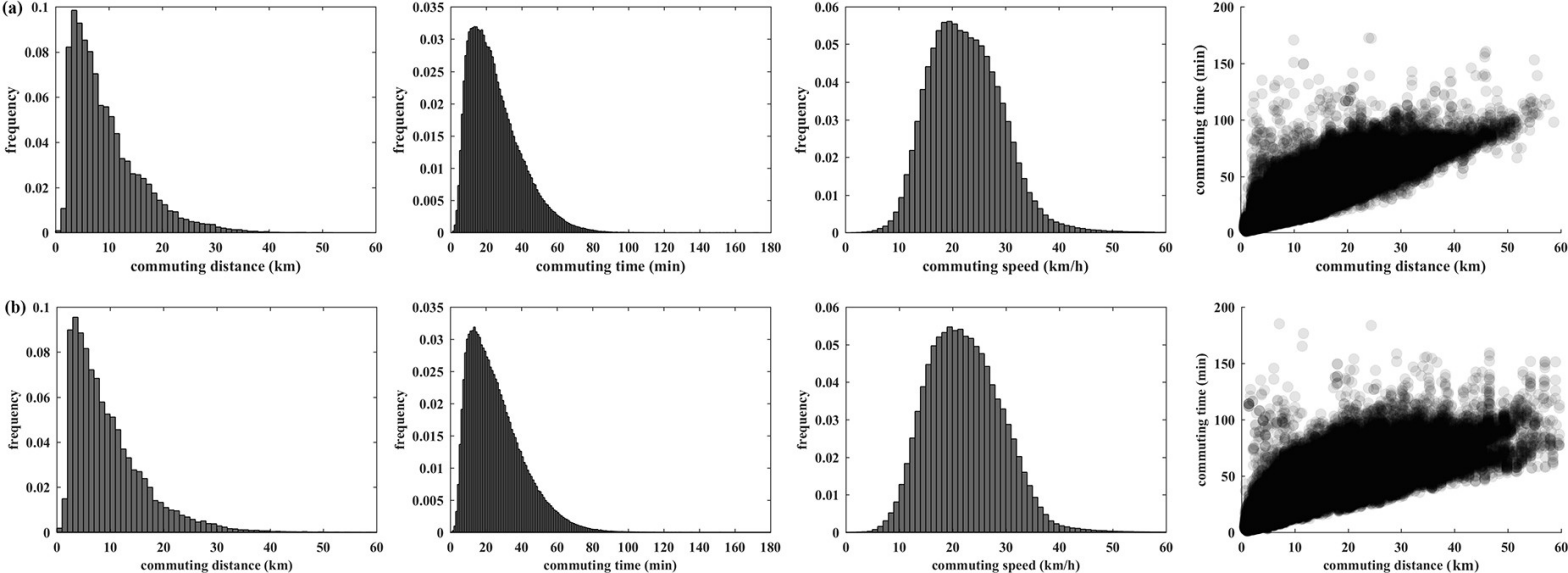
Overall commuting dynamics: (a) 2015, (b) 2016.

This study first analyzed overall commuting dynamics across the whole city. **Fig 3** shows the probability density distribution of one-way commuting time, commuting distance, and commuting speed, along with the relationship between commuting time and distance. Commuting patterns were derived using all commuting trips, including both bus trips and metro trips. In general, similar distribution patterns were observed in commuting distance and time for both study periods. The largest proportion of commuters traveled 3–4 km from home to work in about 10 minutes. After the highest value, the probability density presents a decay pattern that it decreases as commuting time and distance increase. Commuting speed (commuting distance/commuting time) generally follows a normal distribution, with values ranging from 0 to 60 km/h.

**Table 1** shows the average commuting time, distance, and speed before and after rail transit upgrades. On the whole, the average commuting distance for bus trips is considerably shorter than that for metro trips. In 2015, the average commuting distances were about 8 km (bus) and 12 km (metro). The average commuting speed of rail transit was 25 km/h. This is much lower than the running speed of rail because commuting time includes not only in-vehicle travel time but also the waiting time at stations, which is noticeable due to the traffic delay and crowding, especially during peak hours. The average commuting speed of the bus system was around 20 km/h. Because the bus speed is significantly lower than that of rail transit, the bus system mainly serves short- and middle-distance travel while rail transit is developed to serve long-distance trips. However, the two travel modes are competitive in some areas that are covered by both systems.

**Table 1:** Average commuting distance/time/speed across the whole city.

Commuting indicators	2015			2016		
	Bus	Metro	Bus & Metro	Bus	Metro	Bus & Metro
			66.38%: 33.62%			57.41%: 42.59%
**Commuting distance (km)**	7.98	12.48	9.53	7.34	12.14	9.33
**Commuting time (minutes)**	23.49	28.57	25.25	23. 72	28.15	26.40
**Commuting speed (km/h)**	21.21	25.27	22.61	19.41	25.22	21.81

In the short term, no significant changes were found in average commuting time or distance across the whole city. However, the proportion of people traveling by rail transit increased from 33.62% to 42.59% after the opening of new metro lines. This increase is consistent with existent evidence that improvements in rail transit have positive effects on promoting travel modal shifts. However, the average travel speed of bus trips was lower in 2016 than that in the preceding year.

This study further examined changes in the population commuting for different time intervals, i.e. 0–15 minutes, 15–30 minutes, 30–45 minutes, 45–60 minutes, 60–75 minutes, and 75–90 minutes (**Table 2**). Only a small proportion of public transit commuters travel very long distances; most public transit users are short- and middle-distance commuters. The highest proportion ride for an interval of 15–30 minutes, followed by 0–15 minutes. Approximately 70% of public transit commuters live within 30 minutes travel of their workplaces, and over 90% of commuting trips are less than 45 minutes. This finding suggests that most commuters reside in areas where they can travel to their workplaces by public transit within a reasonable commuting time. There was no obvious change in the proportion of very short-distance (within 15 minutes) commutes after the opening of new rail transit lines. The proportion of middle-distance (between 15 and 30 minutes) commutes decreased slightly, while long-distance (more than 30 minutes) commuting trips increased slightly.

**Table 2:** Percentage and cumulative percentage of commuters in different transit time intervals.

Time Interval (minutes)	2015		2016	
	Percentage (%)	Cumulative Percentage (%)	Percentage (%)	Cumulative Percentage (%)
0–15	28.23	28.23	28.38	28.38
15–30	40.52	68.75	39.39	67.77
30–45	20.66	89.41	21.03	88.80
45–60	7.82	97.23	8.14	96.94
60–75	2.19	99.42	2.39	99.33
75–90	0.47	99.89	0.54	99.87
>90	0.11	100	0.13	100
Total	100	-	100	-

In summary, in the very short term after the opening of new metro lines, no considerable changes are observed in average commuting distance and time across the whole city. Does it mean that the opening of new rail transit lines has no impact on urban commuting patterns? If not, how and to what extent rail transit upgrades contribute to commuting dynamics? To answer this question, we further examine the potential effects of rail transit upgrades on the commuting patterns at the region level.

#### Regional changes in accessibility to employment centers

**Fig 4:**
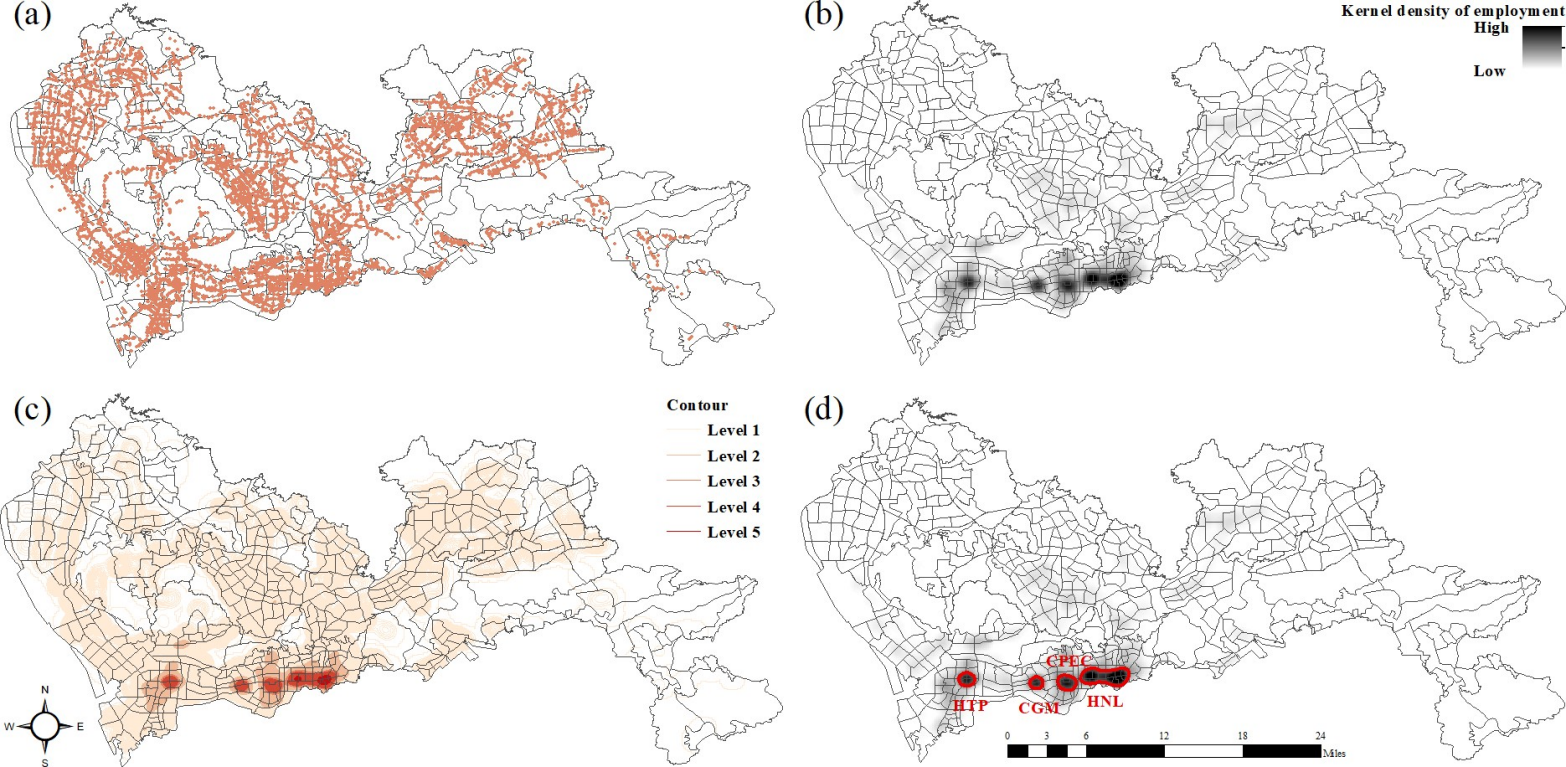
Identification of employment centers: (a) distribution of jobs, (b) kernel density map of employment, (c) contour map of employment, (d) employment centers.

At the region level, we concern about the changes in commuting efficiency brought by rail transit upgrades. The increase in commuting efficiency can lead to better job accessibility. Therefore, we evaluated the effect by examining the changes in job accessibility of residential zones to employment centers. To carry out this analysis, this study first identified the typical employment centers of Shenzhen, areas with high job densities. **Fig 4** presents the analytical framework for identifying employment centers. First, the kernel density method was used to generate the employment density surface of the whole city based on workplace locations derived from smartcard data. To extract high-density employment centers from this surface, we generated a contour map and then classified the density values into five grades using the Jenks classification method. Areas belonging to the fifth grade of the contour map were selected as typical employment centers.

Four typical employment centers were identified (**Fig 4(D)**); all were located in the inner city, suggesting a centralized employment structure for Shenzhen. One employment center is the High-tech Park (HTP) in the Nanshan district, at which the majority of high-tech companies and industries are located. *Chegongmiao* (CGM) is located in the Futian central business district (CBD) and is the most important financial center in Shenzhen. Another typical employment center is the COCO Park and Exhibition Center (CPEC), which is the biggest shopping, business, and entertainment center in the Futian district. Finally, the highest-density employment center is *Huaqiang* North and the *Laojie* area (HNL), which is located in the Luohu CBD. HNL is not only the heart of urban economic activity for Shenzhen but also the most heavily populated area and a cultural hotspot. Ultimately, as the majority of job opportunities are located in the city center, job accessibility can be represented as proximity to employment centers in the inner city.

This study uses commuting time as a proxy for job accessibility. We calculated the average commuting time from each residential zone to each employment center based on individual commuting time and residence. Suppose that *Z*_*ij*_ is the collection of all the commuters who live in TAZ *i* and work in employment center *j*. *AveTime*_*ij*_ represents the commuting time from residential zone TAZ *i* to employment center *j*, and is calculated as follows:

$$Ave{Time}_{ij}=\frac{\sum_{k\in Z_{ij}}t_{k}}{N_{ij}}$$ (1)

where *t*_*k*_ is the commuting time of commuter *k*, and *N*_*ij*_ is the total number of commuters who live in TAZ *i* and work in employment center *j*; *i* =1,…,491; *j* = *HTP*,*CGM*,*CPEC*,*HNL*.

We evaluate average effects of rail transit upgrades on job accessibility of residential zones by using a difference-in-difference (DID) estimator. In this model, we controlled for the district fixed effect and the year fixed effect. The treatment group consisted of residential zones having centroids within 2 km of a station on a newly-opened metro line. The control group consisted of residential zones that are more than 2 km away from the new rail stations. The model is expressed as follows:

$$Ave{Time}_{it}=\beta_{0}+\beta_{1}*{treated}_{i}*{period}_{t}+{\mu_{i}+\lambda_{t}+\varepsilon }_{it}$$ (2)

$${treated}_{i}=\left\{ \begin{array}{l} 1,\;if\;min(distance\langle {taz}_{i},\;stops\rangle )\leq 2km)\\ 0,\;if\;min(distance\langle {taz}_{i},\;stops\rangle )>2km) \end{array}\right.$$ (3)

$${period}_{t}=\left\{ \begin{array}{l} 0,\;if\;t=before\;rail\;transit\;upgrades\\ 1,\;if\;t=after\;rail\;transit\;upgrades \end{array}\right.$$ (4)

where *μ*_*i*_ and *λ*_*t*_ are the district fixed effect and the year fixed effect, respectively; *ε*_*it*_ represents the error term; and *β*_1_ is the average treatment effect of rail transit upgrades on the job accessibility of residential zones. The results of the DID model are shown in **Table 3**.

**Table 3:** Results of DID model for regional commuting time to typical employment centers.

*Employment center*	*Commuting time (min)*	Coef.	Std.Err.	t	P>|t|	[95% Conf. Interval]	
**HTP**	***(constant)***	43.371	0.245	176.76	0.000	42.887	43.854
	treated*period	-2.168	0.887	-2.44	0.015	-3.916	-0.421
	***(period)***						
	***after rail transit upgrades***	4.350	0.666	6.53	0.000	3.038	5.662
	***R***^***2***^	0.240					
**CGM**	***(constant)***	39.194	0.335	116.85	0.000	38.533	39.855
	treated*period	-4.314	1.273	-3.39	0.001	-6.822	-1.806
	***(period)***						
	***after rail transit upgrades***	5.843	1.128	5.18	0.000	3.620	8.066
	***R***^***2***^	0.209					
**CPEC**	***(constant)***	39.705	0.392	101.32	0.000	38.933	40.477
	treated*period	-5.358	1.398	-3.83	0.000	-8.112	-2.604
	***(period)***						
	***after rail transit upgrades***	3.367	1.092	3.08	0.002	1.216	5.517
	***R***^***2***^	0.078					
**HNL**	***(constant)***	40.932	0.225	182.14	0.000	40.490	41.375
	treated*period	-3.250	0.802	-4.05	0.000	-4.828	-1.671
	***(period)***						
	***after rail transit upgrades***	4.578	0.676	6.77	0.000	3.248	5.909
	***R***^***2***^	0.247					

For each employment center, all variables were significantly associated with commuting time. Notably, the coefficient of the average treatment effect is negative and significant at the 0.01 or 0.001 level; this indicates that rail transit upgrades can reduce commuting time from residential zones to employment centers and thereby increase job accessibility. Generally, the average commuting time decreased by 2–5 minutes in the treated area. This result is consistent with existing evidence that improvements in public transport have positive effects, improving commuting efficiency and reducing the travel time of residents who use public transport for commuting [28, 29].

**Fig 5:**
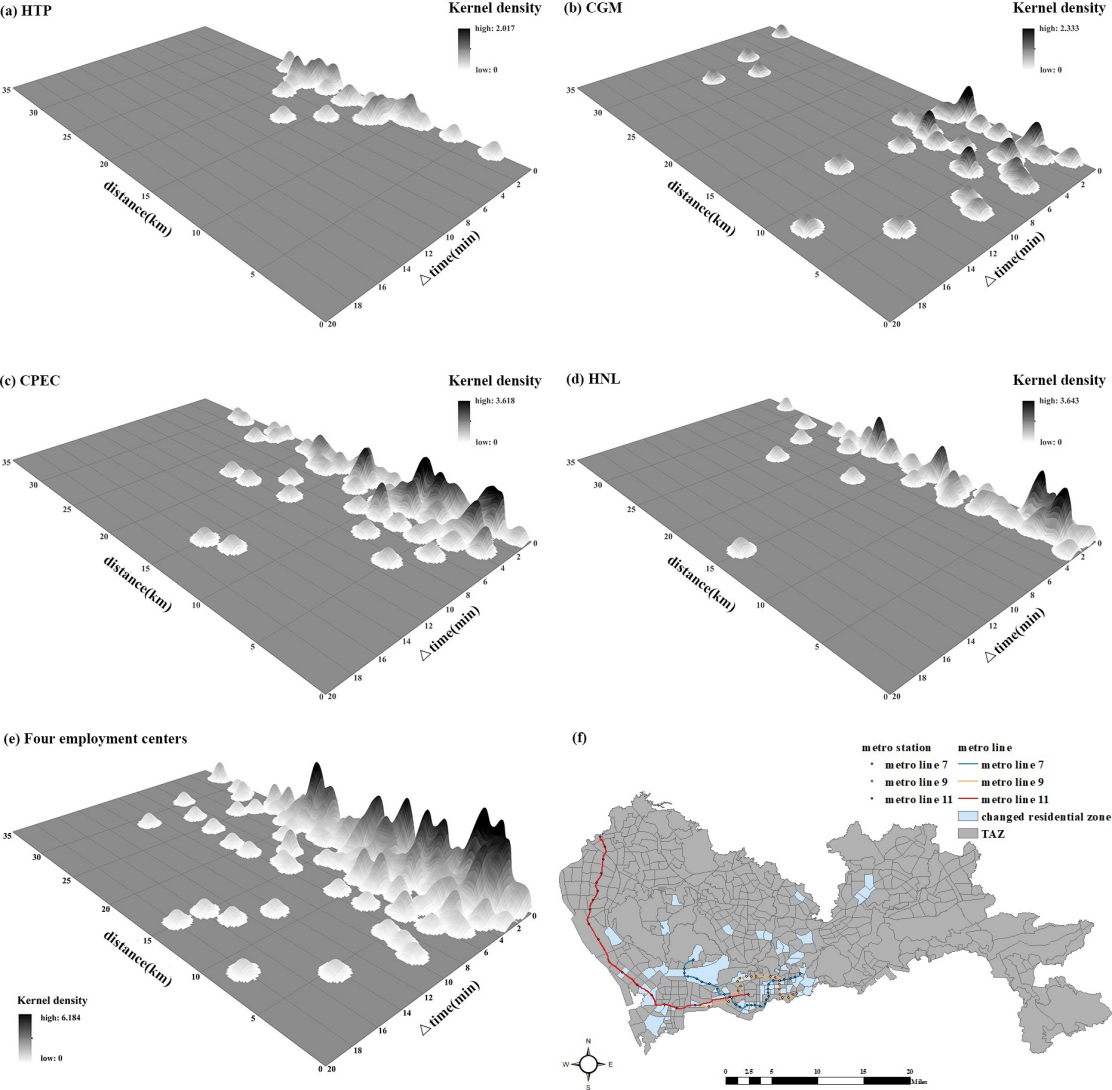
Changes in commuting time to employment centers: (a) HTP, (b) CGM, (c) CPEC, (d) HNL, (e) all typical employment centers, (f) spatial distribution of residential zones that benefited.

To explore which parts of city enjoyed the greatest reductions in commuting time, we calculated the distances from each residential zone to each employment center and the corresponding changes in commuting time. Residential zones with reduced commuting times were used to generate a kernel density trend map, shown in **Fig 5**. The horizontal and vertical coordinates are the reduced time, expressed as Δtime (minutes), and distance to employment center, represented as distance (km), respectively.

The effect of new metro lines on residential zones varies depending on the associated employment center. For the HTP employment center, the residential zones that benefited most are those 7–22 km away (**Fig 5(A)**). In general, the commuting times for these residential zones were reduced by 0–4 minutes. For the CGM and CPEC employment centers, most of the residential zones that benefited are located within 15 km and enjoyed commute improvements of 0–6 minutes (**Fig 5(B) and 5(C)**). Meanwhile, commuting times from residential zones distributed within 20 km of the HNL employment center were only reduced by 0–2 minutes (**Fig 5(D)**). **Fig 5(E)** illustrates the density surface of all benefiting residential zones associated with the four employment centers. The majority of residential zones that benefited are located within 20 km of the employment centers and enjoyed 0–6 minute’s reduction in commuting times. To determine which residential zones enjoyed the greatest improvement, we selected those whose commuting times were reduced by more than 2 minutes, illustrated in **Fig 5(F)**. Most of these residential zones are very close to the new metro stations, which is consistent with the abovementioned finding that rail transit upgrades can strengthen the connections between nearby residential zones and employment centers as well as improve personal mobility.

### Relocation of individual residences and workplaces

In addition to positive effects on improving job accessibility, rail transit can also affect commuting patterns through changing the distribution of residential and workplace locations. Since people prefer to live and work near rail transit services [1], the improvements of urban rail systems may encourage people to relocate or look for job opportunities in the areas served by new rail transit lines [30]. Therefore, to identify the potential impact of rail transit improvement on commuting patterns, changes in commuters’ residence and workplace locations should be considered.

**Table 4** shows overall trends in jobs-housing redistribution across the whole city. Generally, the city evidences a rapid suburbanization trend. The proportion of commuters who lived in the inner city decreased from 49.79% to 41.62%, based on residence locations derived from smartcard data. This indicates that more commuters resided in suburban areas in 2016. Alongside this suburbanization of the population is a trend of shifting employment from the inner city to the suburbs as well; and this employment decentralization is as significant as population suburbanization. In 2015, more than 70% of job opportunities were concentrated in the inner city; but by 2016, the proportion of the population working in the inner city decreased rapidly, from 71.02% to 62.61%. This means that fewer people in the suburbs commute to the city center for work; instead more seek jobs in the suburbs. The percentages of suburb-to-center and suburb-to-suburb commuting trips increased while the within-center commutes decreased greatly. In particular, the proportion of suburb-to-suburb commuters increased significantly, from 25.35% to 33.00%. Taken as a whole, this jobs-housing redistribution presents another manifestation of changing commuting pattern.

**Table 4:** Distribution of commuters in the inner city and suburbs.

Working Population (Percentage %)	Residential Population (Percentage %)					
	2015			2016		
	inner city	suburb	total	inner city	suburb	total
**inner city**	46.16	24.86	71.02	37.23	25.38	62.61
**suburb**	3.63	25.35	28.98	4.39	33.00	37.39
**total**	49.79	50.21	100	41.62	58.38	100

Although rapid suburbanization has affected commuting patterns, e.g. center-to-center commutes changed into suburb-to-suburb commutes, the effects of suburbanization on commuting distance and time are complex. Relocation to suburban districts does not necessarily lead to increases in commuting distance or time. For example, although some commuters resettled in the suburbs, they may find jobs around their new homes instead of remaining with their prior employers. To understand the changes in commuting pattern associated with individual relocation, we extracted commuters who moved or changed their jobs during the study period based on changes in residences or workplaces. Following the study in [31], relocated commuters were classified into three categories: 1) home movers, transit commuters who moved with job unchanged; 2) job hoppers, commuters who changed workplace without changing residence; and 3) job and residence switchers, commuters who changed both workplace and home during the study period. Changes in the distance between home and workplace were calculated for each of these three subgroups.

**Fig 6:**
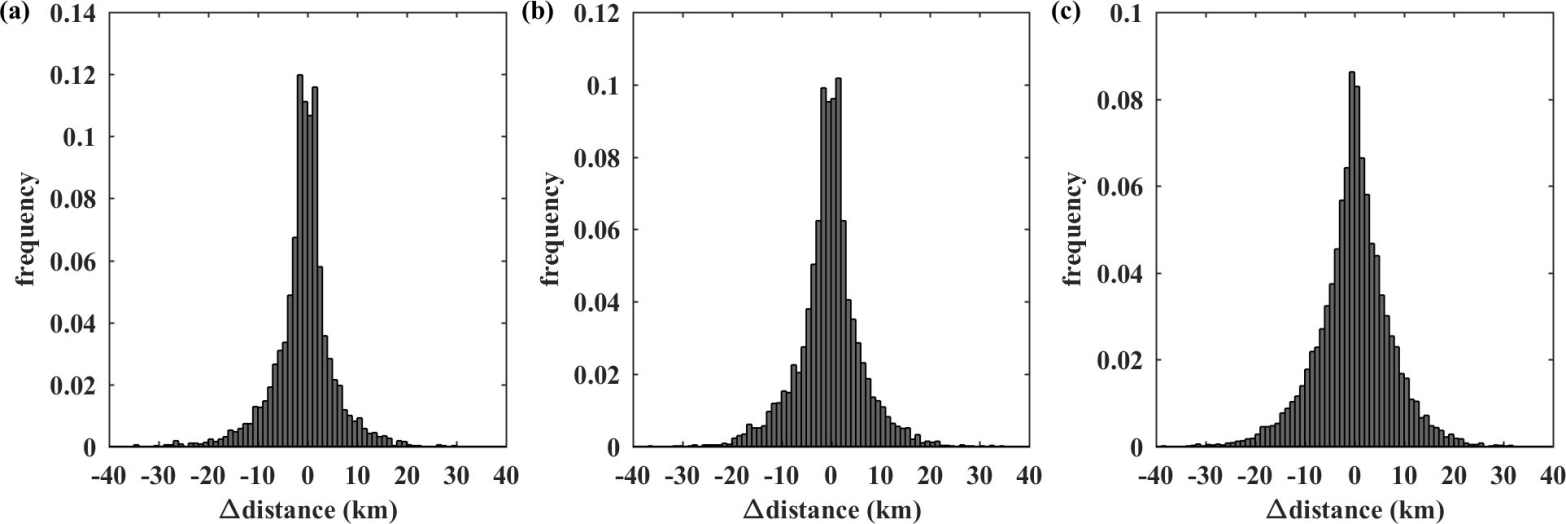
Changes in distances for (a) home movers, (b) job hoppers, and (c) job and residence switchers.

As depicted in **Fig 6**, for each subgroup, the proportion of relocated commuters decreased rapidly with increasing Δdistance. This suggests that most commuters tend to maintain or slightly change the distance between their homes and workplaces, which is consistent with existing evidence that when workers change locations, they prefer commuting zones (i.e., time and distance) similar to their previous commuting zones. These behaviors lead to stable average city-wide commuting time and distance, regardless of high residence and workplace mobility [32]. The symmetrical pattern observed for Δdistance indicates a dynamic balance of jobs-housing redistribution across the whole city. This result is consistent with the findings related to overall dynamics in **Table 1** that there is no considerable change in average commuting distance.

Although the city has witnessed a lot of reallocations over the two years, the large-scale relocations to suburban areas are not only driven by rail transit upgrades. Individual’s relocations are motivated by various factors. In addition to transport systems, people are also influenced and constrained by land use patterns, housing factors, individual socioeconomic attributes, and personal preferences. An empirical study of Shenzhen has found that a large number of public transit commuters moved to suburban areas and urban periphery due to surging housing prices in 2015 [33].

In this study, we are interested in how and to what extent rail transit development contributes to changes in individual commuting patterns. To evaluate this effect, we extracted all relocated individuals (i.e. home movers, job hoppers, job and residence switchers) and applied another DID model. In this model, the commuting distance is considered as the dependent variable, which measures the extent of home-work separation. We defined transit users who commuted by new metro lines (7, 9, and 11) in 2016 as the treatment group. The model is expressed as follows:

$${distance}_{it}=\beta_{0}+\beta_{1}*{treated}_{i}*{period}_{t}+{\mu_{i}+\lambda_{t}+\varepsilon }_{it}$$ (5)

$${treated}_{i}=\left\{ \begin{array}{l} 1,\;if\;{individual}_{i}\in treatment\;group\\ 0,\;if\;i{ndividual}_{i}\in control\;group \end{array}\right.$$ (6)

$${period}_{t}=\left\{ \begin{array}{l} 0,\;if\;t=before\;rail\;transit\;upgrades\\ 1,\;if\;t=after\;rail\;transit\;upgrades \end{array}\right.$$ (7)

Where *distance*_*it*_ is the commuting distance of individual *i* in year *t*. *μ*_*i*_ and *λ*_*t*_ are the individual fixed effect and the year fixed effect, respectively; *ε*_*it*_ is the error term. The results of the DID model for relocated individual commuting distances are given in **Table 5**. The average treatment effect is statistically significant at the 0.001 level and presents a positive association with commuting distance. On average, the commuting distance of relocated individuals increased by 8.6 km due to the opening of new rail transit lines. This suggests that the improvement of rail transit can motivate residents to live farther from their jobs or seek job opportunities farther away from home, exacerbating the separation of residence and workplace.

**Table 5:** Results of DID model for relocated individual commuting distance.

*Distance (km)*	Coef.	Robust Std.Err.	t	P>|t|	[95% Conf. Interval]	
***(constant)***	11.249	0.019	580.80	0.000	11.211	11.287
treated*period	8.568	0.399	21.470	0.000	7.786	9.350
***(period)***						
***after rail transit upgrades***	-0.624	0.039	-16.05	0.000	-0.700	-0.548
***R***^***2***^	0.013					

These effects can be explained by two potential factors. On factor is the high commuting efficiency that results from rail transit upgrades, which reduces the cost of commuting to employment centers and other destinations, in turn, increases the attractiveness of new station areas, causing population relocation to zones near the new rail transit lines. Therefore, the opening of suburban metro lines would incite people to relocate into less central areas, leading to the increase in their commuting distances.

Moreover, rail transit upgrades also influence commuting patterns through effects on the real estate values of neighboring zones. According to the theory of urban spatial equilibrium based on the trade-off between accessibility and cost of space, reduced travel costs and rising demand to live close to new transit stations are expected to increase housing costs in the beneficiary areas [34, 35]. To illustrate these effects, we applied a DID model to the average housing unit prices of residential zones before and after rail transit upgrades derived from a real estate rental and sales service platform in China, *Anjuke* (https://shenzhen.anjuke.com/). The DID model and results are provided in **S1 Appendix**. The results demonstrate that the rail transit upgrades considerably increased housing prices in treatment areas close to the new rail transit lines. Consequently, people who cannot afford the extremely high housing costs in central areas will reduce rents by changing locations, moving to the suburban zones along new rail transit lines. As a result, rail transit expansion increases population density in the suburbs and commuting distances of relocated individuals.

## Discussion

In this study, we conducted the analysis at three levels (i.e. city-region-individual) and drawn the following findings: 1) The average commuting distance and time did not change significantly across the whole city in the study period. 2) The opening of new rail transit lines reduces the commuting time of new station zones to typical employment centers. 3) Rail transit upgrades increase individual commuting distances by influencing residence and job relocations. Why does the overall commuting time remain stable while new rail transit lines reduce the commuting time in the treatment zones? Why does the overall commuting distance have no obvious change while new rail transit lines increase individual commuting distances? The overall commuting dynamics are the outcomes of various influencing factors, the impact of rail transit cannot be intuitively reflected in the overall commuting patterns. In order to examine the changes in commuting patterns brought by only rail transit upgrades, we conducted the analysis at the region level and individual level. Therefore, the influencing results of rail transit upgrades on regions and individuals may not be consistent with the overall commuting dynamics.

Moreover, as we aim to study the impacts on commuting dynamics from different perspectives (i.e. commuting efficiency and jobs-housing separation), the region level analysis and individual level analysis focus on different groups. The target group for region level analysis can be considered as the commuters who live in the treated zones and work in the employment centers. New rail transit lines increased their commuting efficiency and reduce their commuting time with constant commuting distance. The target group for individual level analysis is the commuters who originally lived in the control area and relocated to the treated area. After relocations, their commuting distance increased. For these commuters, their commuting time may increase as well. The two effects will jointly affect the overall commuting dynamics. Therefore, the respective result of region level analysis or individual level analysis may be inconsistent with the overall commuting patterns.

**Fig 7:**
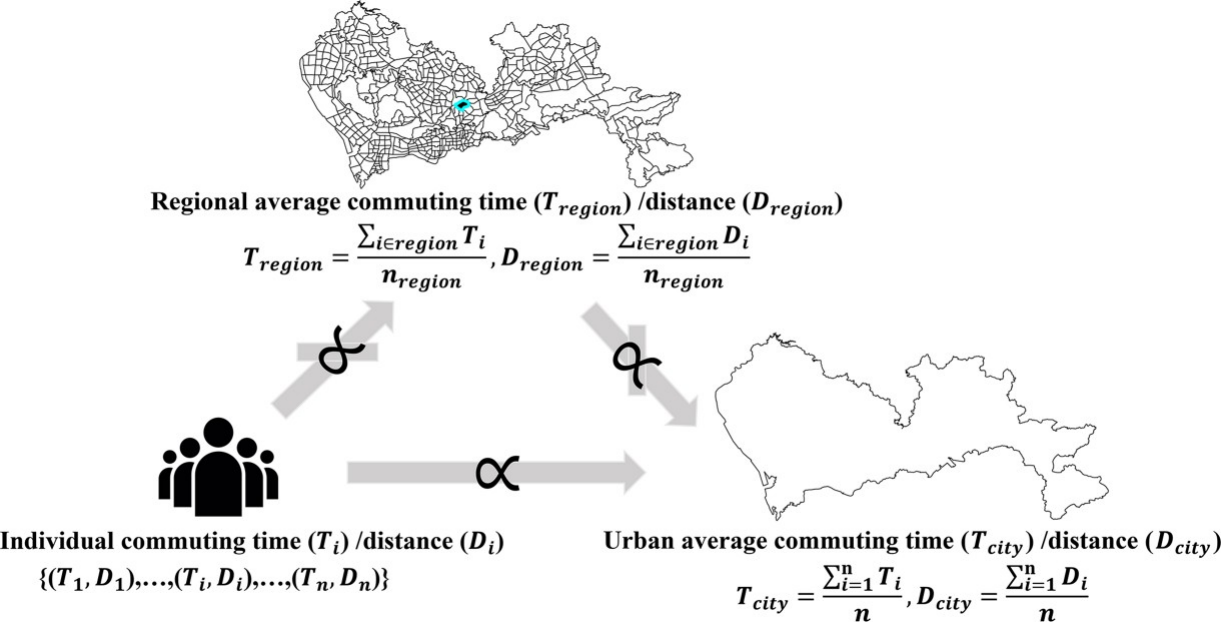
Relationship between individual, region and urban commuting indicators.

In addition, the analysis at the region level and individual level are based on different analysis units, the increase (decrease) in individual commuting indicators may not result in the increase (decrease) in regional commuting indicators. We illustrate the relationships between the commuting indicators of three levels, as displayed in **Fig 7**. The commuting time and distance of individual *i* are expressed as *T*_*i*_ and *D*_*i*_, then the regional average commuting time and distance are $T_{region}=\frac{\sum_{i\in region}T_{i}}{n_{region}}\;and\;D_{region}=\frac{\sum_{i\in region}D_{i}}{n_{region}}$, where *n*_*region*_ is the number of commuters in the region. The average commuting time and distance at the city level are defined as $T_{city}=\frac{\sum_{i=1}^{n}T_{i}}{n}\;and\;D_{city}=\frac{\sum_{i=1}^{n}D_{i}}{n}$, where *n* is the number of commuters in the city.

**Fig 8:**
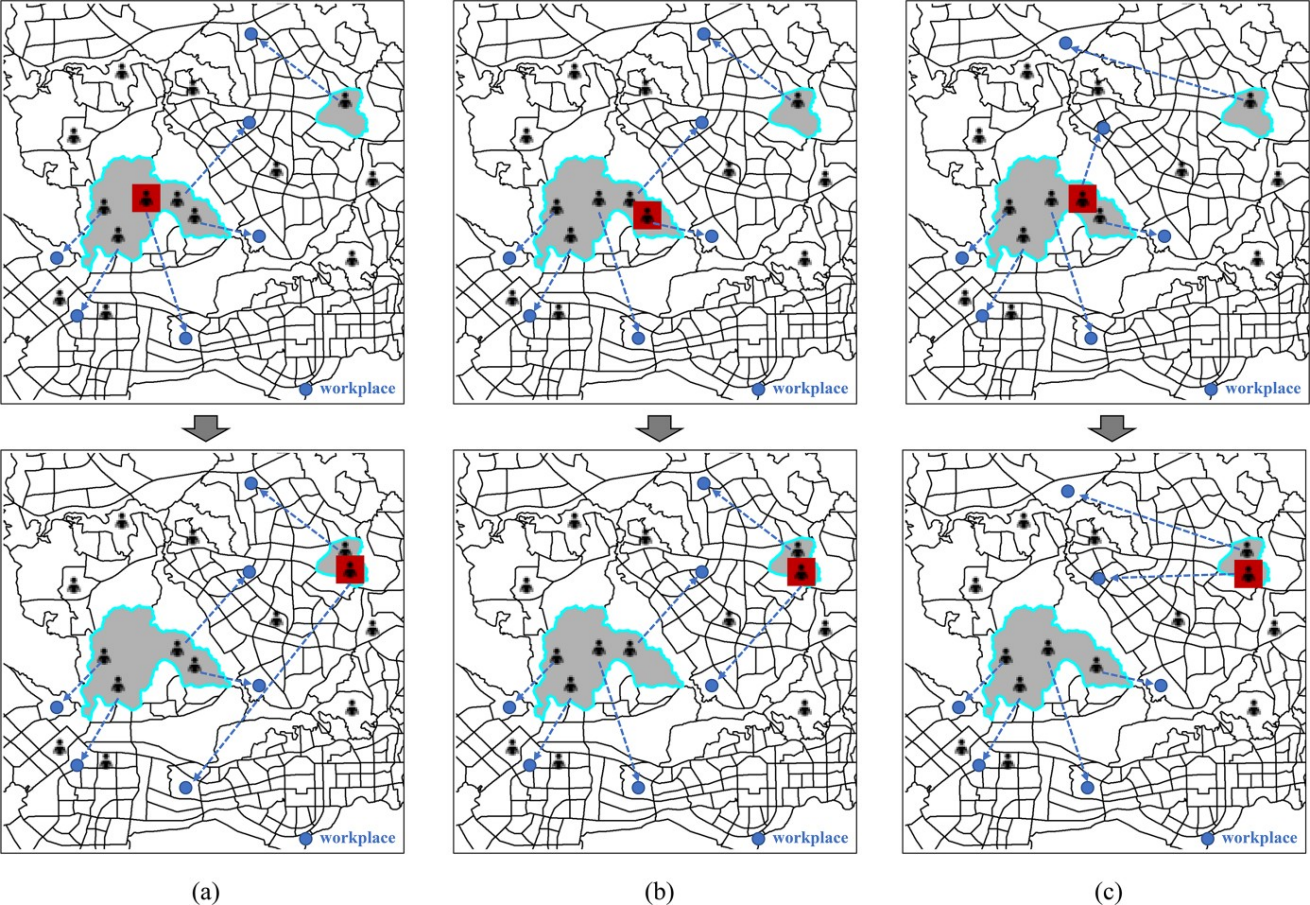
Illustrations of the effect of individual’s relocation on regional commuting indicators.

Firstly, changes in individual’s commuting time/distance can generate a variety of possible outcomes on the regional average commuting time/distance. Increase in individual’s commuting time/distance may not lead to an increase in average commuting time/distance in the original residential area or resettled region. It depends on the numerical relationship (i.e. larger, smaller and equal) between individual’s commuting time/distance and regional average commuting time/distance. **Fig 8** illustrates three examples that describe different situations. Although all the highlighted commuters moved from one region to another and the individual’s commuting time/distance increased, the effects of the relocations on regional commuting patterns vary. In **Fig 8(A)**, the commuter’s previous and present commuting time/distance are respectively larger than the average commuting time/distance in the original residential region and relocated region, therefore the relocation would lead to the reduction in average commuting time/distance in the original region while increase in the resettled region. For the commuter in **Fig 8(B)**, he used to live close to where he works and then moved to a farther place, which would lead to an increase in the average commuting time/distance in both his original and resettled regions. Inversely, the commuter’s relocation behavior in **Fig 8(C)** would lead to the reduction in commuting time/distance in both regions.

The increase in individual’s commuting time/distance is positively associated with the increase in overall commuting time/distance. Regardless of other unknown factors influencing commuting dynamics, the stable overall commuting distance may be attributed to the relatively short study period. The data used in this study were collected over a period of only five months after the opening of one new metro line (No. 11) and one month for the other two (No. 7 and 9). Although new rail transit can motivate people to relocate, only a very small number of commuters were affected over a period of a few months because residential mobility is time-consuming and complicated. Compared with the total millions of transit users, the population of relocated commuters is not large enough to make a noticeable difference in overall commuting distance across the whole city.

Two factors may contribute to the stable average commuting time. The first is the decrease in commuting speed of bus trips alongside the increased speed of new metro systems (see **Table 1**). The second possible reason is the “rebound effect”, in which increased speed due to rail transit upgrades reduces commuting cost to make people commute farther, which has been demonstrated in this study. Although improvement of rail transit reduces the travel time of some commuters who live in the treatment area, the greater distance has an opposing effect on the overall commuting time. This shed light on the importance of examining commuting dynamics in response to rail transit upgrades at multiple levels.

In the long term, an increase in average commuting distance is expected across the whole city. General demand to live close to efficient public transit will increase local demand for living in the new rail transit zones, thereby attracting more people to relocate into the outer metro zones. This assumption is supported by existing evidence that the expansion of urban rail transit causes suburbanization [8]. Furthermore, rail transit upgrades affect commuting patterns by increasing real estate values; namely, high housing price and rents in the central zones motivate people to relocate to suburban metro zones, leading to increases in their commuting distances.

However, the change in commuting time is unpredictable. In addition to the “rebound effect”, population suburbanization and employment decentralization also play vital roles in changing commuting time. Therefore, changes in commuting time and distance might be not consistent between different development contexts and land use patterns. For instance, existing research shows that commuting time remains relatively stable despite an increase in average commuting distance [36].

## Conclusions

This study utilized individual-based public transit smartcard data to analyze urban commuting dynamics in response to rail transit upgrades. Using personal commuting information (i.e. residence, workplace, commuting time, and commuting distance) derived from smartcard data before and after the opening of new rail transit lines, this study proposed a multi-level analytical framework to explore changes in commuting patterns, including city level, region level and individual level. Difference-in-difference models were adopted to evaluate the effects of the rail transit system on commuting patterns. This study has demonstrated that improvement of rail transit had positive effects on improving the job accessibility of residential zones and reducing commuting time to employment centers. Meanwhile, new rail transit also promoted individual relocation, which increased the distances between residences and workplaces.

Although this study examined only the short-term effects of rail transit improvement, the proposed analytical framework is meaningful in response to the illustration of dynamic changes and exploration of the trend. In the future, we will examine the long-term effects of rail transit upgrades on urban commuting behaviors by collecting data across multiple years. Furthermore, rail transit improvement not only influences the jobs-housing relationships of public transit commuters but also has an impact on the commuting behaviors of all workers, especially in promoting modal shift; this is another subject for future research. In general, the improvement of rail transit will have a positive effect on job accessibility. These improvements enhance personal mobility and regional connectivity, which are helpful in promoting rail transit use and reducing automobile dependence [9]; thus, the jobs-housing relationships of all workers and the urban spatial arrangement can be changed by transit development. However, these impacts cannot be identified from transit users’ travel behaviors alone; therefore, the effect of rail transit improvement on all workers was not analyzed in this study. To determine the overall effect on jobs-housing mismatch for the city, a longitudinal panel of household travel survey data is needed. This issue may be explored in a future study when such data is available.

## Supporting information

S1 Appendix A difference-in-difference model for estimating the impact of rail transit upgrades on housing prices. (PDF)

S1 File Housing price data. (PDF)
