# Supplementary material for: Urban commuting dynamics in response to public transit upgrades: A big data approach
Source: PLoS One. 2019 Oct 17;14(10):e0223650. doi: 10.1371/journal.pone.0223650 (PMC6797187; doi:10.1371/journal.pone.0223650)
Supplement: S1 Appendix — (PDF) [file pone.0223650.s001.pdf]

S1 Appendix. A difference-in-difference model for estimating the impact of rail transit upgrades on housing prices.

We defined residential zones within 2 km of a new transit station as the treatment group. The dependent variable is the average housing unit price per zone, represented as *hp*. The model is expressed as follows.

$$hp_{it} = \beta_0 + \beta_1 * treated_i * period_t + \mu_i + \lambda_t + \varepsilon_{it} \quad (1)$$

$$treated_i = \begin{cases} 1, & if \min\left(distance\langle taz_i,\ stops\rangle\right) \le 2km \\ 0, & if \min\left(distance\langle taz_i,\ stops\rangle\right) > 2km \end{cases} \quad (2)$$

$$period_t = \begin{cases} 0, & if\ t = before\ rail\ transit\ upgrades \\ 1, & if\ t = after\ rail\ transit\ upgrades \end{cases} \quad (3)$$

hp_{it}-average housing unit price of TAZ *i* in the period *t*, $i = 1, \ldots, 491$;

β_1-the average treatment effect of rail transit upgrades on housing unit price of residential zones;

μ_i-district fixed effect;

λ_t-year fixed effect;

ε_{it}-error term.

The results of the DID model are given in the **Table S1**.

Table S1. Results of DID model for average housing unit price.

Housing price (yuan/m^2)	Coef.	Std.Err.	t	P>|t|	[95% Conf. Interval]	
(constant)	27577.11	439.21	62.79	0.000	26714.14	28440.08
*treated*period*	7817.90	1756.10	4.45	0.000	4367.47	11268.32
(period)						
after rail transit upgrades	27475.58	1177.70	23.33	0.000	25161.61	29789.56
R^2	0.727					
