# Supplementary material for: Urban commuting dynamics in response to public transit upgrades: A big data approach
Source: PLoS One. 2019 Oct 17;14(10):e0223650. doi: 10.1371/journal.pone.0223650 (PMC6797187; doi:10.1371/journal.pone.0223650)
Supplement: S1 File — (PDF) [file pone.0223650.s002.pdf]

tazid	housingprice(yuan/m²)	treated	period	treated*period
40030	78323	0	0	0
40030	62389	0	1	0
40066	16028	0	0	0
40066	33342	0	1	0
40018	18394	0	0	0
40018	35658	0	1	0
40071	22276	0	0	0
40071	38461	0	1	0
40039	38123	0	0	0
40039	49259	0	1	0
60031	12848	0	0	0
60031	31463	0	1	0
60036	6634	0	0	0
60036	32046	0	1	0
40006	28391	0	0	0
40006	68235	0	1	0
40068	19796	0	0	0
40068	56773	0	1	0
10176	31162	0	0	0
10176	44891	0	1	0
10178	21302	0	0	0
10178	58464	0	1	0
10165	31997	0	0	0
10165	53768	0	1	0
30302	34924	1	0	0
30302	64701	1	1	1
30345	36074	1	0	0
30345	62174	1	1	1
30281	36462	1	0	0
30281	65559	1	1	1
50247	36594	1	0	0
50247	76148	1	1	1
50207	23427	1	0	0
50207	55952	1	1	1
50025	59782	1	0	0
50025	95551	1	1	1
10193	22521	1	0	0
10193	57926	1	1	1
10248	21218	1	0	0
10248	45553	1	1	1
10189	27005	0	0	0
10189	50760	0	1	0
10179	21574	0	0	0
10179	50556	0	1	0
10011	22046	1	0	0
10011	50230	1	1	1
10113	24001	1	0	0
10113	40561	1	1	1
30306	24381	1	0	0
30306	56663	1	1	1
10042	20225	1	0	0

10042	53468	1	1	1
10155	27290	1	0	0
10155	50433	1	1	1
10166	23432	1	0	0
10166	40542	1	1	1
10215	21057	1	0	0
10215	110791	1	1	1
10006	25476	1	0	0
10006	40925	1	1	1
20084	42391	1	0	0
20084	7385	1	1	1
20014	26321	1	0	0
20014	53588	1	1	1
20007	31785	1	0	0
20007	60518	1	1	1
30216	32804	1	0	0
30216	56657	1	1	1
10152	29217	1	0	0
10152	54916	1	1	1
10244	19282	1	0	0
10244	39820	1	1	1
10192	21610	1	0	0
10192	41392	1	1	1
10010	19818	1	0	0
10010	39927	1	1	1
10115	21934	1	0	0
10115	58880	1	1	1
10181	24077	1	0	0
10181	51455	1	1	1
10199	28065	1	0	0
10199	42857	1	1	1
10169	67721	1	0	0
10169	84034	1	1	1
10036	24534	1	0	0
10036	60000	1	1	1
20004	25351	1	0	0
20004	46028	1	1	1
20037	40413	1	0	0
20037	95710	1	1	1
20234	40642	1	0	0
20234	82609	1	1	1
20036	33865	1	0	0
20036	95412	1	1	1
20032	25765	1	0	0
20032	45993	1	1	1
20109	27577	1	0	0
20109	48800	1	1	1
20108	30010	1	0	0
20108	61538	1	1	1
20117	34508	1	0	0
20117	93971	1	1	1
20107	31352	1	0	0

20107	60935	1	1	1
20252	62411	1	0	0
20252	107131	1	1	1
20096	37087	1	0	0
20096	72500	1	1	1
20087	25162	1	0	0
20087	52335	1	1	1
20138	28256	1	0	0
20138	77607	1	1	1
20259	59025	1	0	0
20259	174597	1	1	1
20074	44781	1	0	0
20074	100803	1	1	1
20073	39901	1	0	0
20073	96568	1	1	1
20291	34244	1	0	0
20291	69788	1	1	1
30033	31352	1	0	0
30033	60708	1	1	1
30035	87169	1	0	0
30035	156558	1	1	1
20062	28741	1	0	0
20062	54369	1	1	1
20211	30127	1	0	0
20211	70769	1	1	1
20167	66314	1	0	0
20167	126970	1	1	1
20075	46823	1	0	0
20075	83592	1	1	1
30121	33740	0	0	0
30121	118417	0	1	0
30132	37892	0	0	0
30132	9280	0	1	0
30174	25892	0	0	0
30174	56637	0	1	0
20022	33199	1	0	0
20022	52344	1	1	1
20269	34771	1	0	0
20269	81927	1	1	1
20274	29839	1	0	0
20274	55263	1	1	1
30317	53359	0	0	0
30317	143161	0	1	0
30077	41451	1	0	0
30077	95781	1	1	1
30318	55670	0	0	0
30318	112515	0	1	0
30200	63054	0	0	0
30200	102898	0	1	0
30055	42763	0	0	0
30055	71638	0	1	0
30296	30477	0	0	0

30296	118231	0	1	0
30298	40776	0	0	0
30298	81765	0	1	0
30230	57362	0	0	0
30230	178764	0	1	0
30371	56516	1	0	0
30371	175543	1	1	1
30148	61278	1	0	0
30148	170767	1	1	1
30066	45067	0	0	0
30066	132716	0	1	0
30071	46872	0	0	0
30071	144589	0	1	0
30118	52483	0	0	0
30118	107133	0	1	0
30116	48031	1	0	0
30116	124133	1	1	1
30380	53394	1	0	0
30380	145588	1	1	1
30399	58566	1	0	0
30399	112984	1	1	1
30094	41626	1	0	0
30094	69822	1	1	1
30115	40777	1	0	0
30115	97139	1	1	1
30084	37551	0	0	0
30084	83117	0	1	0
30093	23541	1	0	0
30093	190140	1	1	1
30312	32017	0	0	0
30312	64341	0	1	0
30159	56640	0	0	0
30159	77206	0	1	0
30284	30921	0	0	0
30284	69778	0	1	0
30063	30431	0	0	0
30063	86667	0	1	0
30425	50926	0	0	0
30425	112319	0	1	0
30379	24012	0	0	0
30379	55172	0	1	0
30426	30095	0	0	0
30426	85470	0	1	0
30182	33776	0	0	0
30182	75294	0	1	0
30291	31202	0	0	0
30291	91666	0	1	0
30290	30690	0	0	0
30290	75852	0	1	0
30072	32327	0	0	0
30072	59949	0	1	0
30087	40071	1	0	0

30087	77786	1	1	1
30088	32172	0	0	0
30088	59756	0	1	0
30089	34079	0	0	0
30089	78182	0	1	0
30180	36540	0	0	0
30180	70765	0	1	0
30085	30633	1	0	0
30085	77116	1	1	1
30186	38281	1	0	0
30186	75683	1	1	1
30322	30370	1	0	0
30322	44672	1	1	1
30187	25240	1	0	0
30187	66899	1	1	1
30097	34717	1	0	0
30097	62116	1	1	1
30265	30426	1	0	0
30265	60903	1	1	1
30324	29991	1	0	0
30324	70246	1	1	1
30414	29822	1	0	0
30414	48276	1	1	1
30175	26157	1	0	0
30175	53046	1	1	1
30106	34106	1	0	0
30106	61005	1	1	1
30191	33743	1	0	0
30191	92671	1	1	1
30128	29795	1	0	0
30128	61012	1	1	1
30305	37754	1	0	0
30305	79182	1	1	1
30287	32167	1	0	0
30287	82133	1	1	1
30413	27692	1	0	0
30413	53763	1	1	1
30010	65363	1	0	0
30010	154201	1	1	1
20099	41203	1	0	0
20099	77098	1	1	1
20287	37126	1	0	0
20287	76271	1	1	1
20006	27158	1	0	0
20006	46154	1	1	1
20226	30721	1	0	0
20226	53745	1	1	1
20086	33216	1	0	0
20086	58310	1	1	1
20237	31489	1	0	0
20237	73598	1	1	1
20080	24956	1	0	0

20080	43064	1	1	1
20053	33410	1	0	0
20053	80218	1	1	1
20265	38176	1	0	0
20265	83327	1	1	1
20054	47467	1	0	0
20054	117967	1	1	1
20121	38079	1	0	0
20121	80005	1	1	1
20058	28808	1	0	0
20058	48187	1	1	1
20267	28661	1	0	0
20267	60534	1	1	1
20230	33602	1	0	0
20230	64238	1	1	1
20186	22763	1	0	0
20186	66358	1	1	1
20089	26707	1	0	0
20089	54683	1	1	1
20009	26908	1	0	0
20009	48781	1	1	1
20011	34615	1	0	0
20011	59291	1	1	1
20029	23536	1	0	0
20029	57692	1	1	1
10030	18225	1	0	0
10030	40478	1	1	1
90022	27423	0	0	0
90022	46376	0	1	0
90004	53173	0	0	0
90004	107470	0	1	0
90144	21730	0	0	0
90144	34906	0	1	0
90118	18904	0	0	0
90118	41058	0	1	0
20169	44189	1	0	0
20169	127958	1	1	1
20247	63057	1	0	0
20247	73150	1	1	1
90053	36161	0	0	0
90053	80717	0	1	0
90175	27342	0	0	0
90175	65409	0	1	0
90176	25838	0	0	0
90176	48942	0	1	0
20046	33023	1	0	0
20046	57609	1	1	1
20281	41166	1	0	0
20281	96983	1	1	1
20145	28424	1	0	0
20145	59080	1	1	1
20147	24375	1	0	0

20147	74967	1	1	1
20283	39038	1	0	0
20283	72114	1	1	1
20042	41041	1	0	0
20042	83529	1	1	1
20114	27820	1	0	0
20114	69722	1	1	1
90043	16752	0	0	0
90043	65705	0	1	0
90042	24179	0	0	0
90042	52961	0	1	0
90006	38944	0	0	0
90006	38166	0	1	0
90178	23076	0	0	0
90178	56338	0	1	0
90051	21143	0	0	0
90051	41552	0	1	0
90099	19910	0	0	0
90099	42551	0	1	0
90167	27059	0	0	0
90167	41067	0	1	0
90172	24394	0	0	0
90172	44817	0	1	0
90225	25513	0	0	0
90225	53123	0	1	0
90267	26445	0	0	0
90267	54254	0	1	0
90273	31881	0	0	0
90273	90877	0	1	0
90098	21094	0	0	0
90098	38532	0	1	0
90265	19975	0	0	0
90265	52122	0	1	0
90264	22890	0	0	0
90264	44115	0	1	0
90189	21379	0	0	0
90189	40937	0	1	0
30224	27938	1	0	0
30224	59348	1	1	1
50558	20485	1	0	0
50558	43832	1	1	1
50186	23537	0	0	0
50186	55504	0	1	0
50173	21045	0	0	0
50173	77328	0	1	0
50017	27692	0	0	0
50017	58735	0	1	0
50343	26980	0	0	0
50343	49620	0	1	0
50031	21961	0	0	0
50031	51634	0	1	0
50409	26093	0	0	0

50409	49945	0	1	0
50512	19813	1	0	0
50512	43646	1	1	1
50511	16292	0	0	0
50511	43998	0	1	0
70106	4697	0	0	0
70106	29904	0	1	0
50170	22281	0	0	0
50170	49826	0	1	0
50506	15762	0	0	0
50506	38460	0	1	0
50123	18895	0	0	0
50123	20752	0	1	0
50212	78852	0	0	0
50212	90865	0	1	0
50248	15719	0	0	0
50248	39535	0	1	0
50013	23409	0	0	0
50013	49032	0	1	0
50185	15261	0	0	0
50185	38889	0	1	0
50444	24523	0	0	0
50444	53392	0	1	0
50188	17731	0	0	0
50188	7887	0	1	0
50306	26425	1	0	0
50306	55841	1	1	1
50177	17933	0	0	0
50177	50484	0	1	0
50443	22687	0	0	0
50443	48918	0	1	0
50206	20436	0	0	0
50206	42074	0	1	0
50456	73236	1	0	0
50456	86923	1	1	1
50062	27149	1	0	0
50062	61459	1	1	1
50042	28032	1	0	0
50042	57189	1	1	1
50430	44641	1	0	0
50430	82148	1	1	1
50043	32302	1	0	0
50043	64765	1	1	1
50050	27656	1	0	0
50050	56585	1	1	1
50041	31262	1	0	0
50041	63564	1	1	1
50457	37937	1	0	0
50457	75840	1	1	1
50215	28764	1	0	0
50215	57307	1	1	1
50213	30813	1	0	0

50213	64845	1	1	1
50395	28567	1	0	0
50395	55405	1	1	1
50405	24553	1	0	0
50405	52053	1	1	1
50018	31570	1	0	0
50018	60461	1	1	1
50184	20379	0	0	0
50184	45670	0	1	0
50459	31905	1	0	0
50459	72017	1	1	1
50417	16355	0	0	0
50417	38034	0	1	0
50037	24286	1	0	0
50037	50135	1	1	1
50427	25225	0	0	0
50427	53892	0	1	0
50020	23234	0	0	0
50020	51987	0	1	0
50175	17290	0	0	0
50175	31579	0	1	0
50410	27917	0	0	0
50410	55870	0	1	0
50168	21117	0	0	0
50168	57881	0	1	0
50426	23423	1	0	0
50426	39192	1	1	1
50028	26747	1	0	0
50028	53441	1	1	1
50458	57557	1	0	0
50458	90171	1	1	1
50027	44924	1	0	0
50027	97709	1	1	1
50245	22409	1	0	0
50245	64103	1	1	1
50167	39344	1	0	0
50167	63811	1	1	1
50313	23691	1	0	0
50313	48638	1	1	1
50044	37551	1	0	0
50044	74337	1	1	1
50378	21641	0	0	0
50378	39640	0	1	0
50407	22823	0	0	0
50407	40121	0	1	0
50420	23571	0	0	0
50420	47648	0	1	0
50519	22110	0	0	0
50519	55680	0	1	0
50218	21999	0	0	0
50218	42438	0	1	0
50477	5436	0	0	0

50477	9000	0	1	0
50568	18313	1	0	0
50568	35084	1	1	1
50508	23434	1	0	0
50508	36120	1	1	1
50274	9152	1	0	0
50274	40834	1	1	1
50091	12597	1	0	0
50091	35000	1	1	1
50321	9357	1	0	0
50321	14615	1	1	1
50094	18041	1	0	0
50094	39474	1	1	1
50228	16395	1	0	0
50228	31232	1	1	1
50113	15193	1	0	0
50113	35289	1	1	1
70026	9859	0	0	0
70026	24793	0	1	0
50528	22989	0	0	0
50528	34752	0	1	0
30251	20673	1	0	0
30251	54348	1	1	1
30327	27464	1	0	0
30327	52432	1	1	1
30212	34332	1	0	0
30212	72279	1	1	1
30231	30114	1	0	0
30231	63353	1	1	1
30205	36276	1	0	0
30205	68395	1	1	1
30402	32626	1	0	0
30402	64722	1	1	1
30278	31473	1	0	0
30278	59086	1	1	1
30335	34639	0	0	0
30335	76035	0	1	0
30326	25393	0	0	0
30326	42318	0	1	0
30024	27009	1	0	0
30024	57730	1	1	1
30019	23561	1	0	0
30019	50000	1	1	1
30275	9448	1	0	0
30275	85435	1	1	1
30258	31564	1	0	0
30258	73529	1	1	1
30351	26356	0	0	0
30351	57006	0	1	0
30338	43914	0	0	0
30338	91157	0	1	0
30041	26507	1	0	0

30041	66932	1	1	1
30241	24128	1	0	0
30241	56070	1	1	1
20170	43211	1	0	0
20170	44818	1	1	1
30042	55637	1	0	0
30042	71574	1	1	1
90182	28905	0	0	0
90182	61798	0	1	0
90168	35527	0	0	0
90168	70356	0	1	0
90275	26219	0	0	0
90275	55848	0	1	0
90274	29888	0	0	0
90274	57935	0	1	0
90181	30129	0	0	0
90181	72306	0	1	0
90096	20387	0	0	0
90096	34769	0	1	0
70053	5951	0	0	0
70053	39110	0	1	0
40062	15406	0	0	0
40062	69939	0	1	0
40008	20707	0	0	0
40008	34636	0	1	0
10096	26289	0	0	0
10096	47189	0	1	0
60312	23222	0	0	0
60312	44516	0	1	0
10098	23258	0	0	0
10098	149533	0	1	0
10235	32473	0	0	0
10235	71405	0	1	0
60842	15793	0	0	0
60842	32972	0	1	0
60337	15761	0	0	0
60337	32752	0	1	0
90088	28710	0	0	0
90088	44954	0	1	0
60345	7150	0	0	0
60345	45614	0	1	0
90030	21018	0	0	0
90030	41918	0	1	0
10224	33273	0	0	0
10224	53551	0	1	0
10134	22683	0	0	0
10134	58377	0	1	0
10100	24624	0	0	0
10100	56412	0	1	0
10050	21621	0	0	0
10050	54595	0	1	0
60167	15999	0	0	0

60167	36308	0	1	0
60489	23750	0	0	0
60489	41563	0	1	0
60190	22519	0	0	0
60190	40504	0	1	0
60480	22219	0	0	0
60480	40764	0	1	0
60813	19269	0	0	0
60813	38773	0	1	0
60745	20536	0	0	0
60745	38889	0	1	0
60017	14038	0	0	0
60017	31794	0	1	0
60210	18790	0	0	0
60210	38870	0	1	0
60716	14065	0	0	0
60716	35011	0	1	0
20205	34619	1	0	0
20205	72560	1	1	1
10141	21824	1	0	0
10141	53280	1	1	1
60559	22928	0	0	0
60559	44736	0	1	0
60731	19522	0	0	0
60731	36206	0	1	0
60188	15663	0	0	0
60188	43893	0	1	0
20219	26202	1	0	0
20219	75704	1	1	1
10112	37785	1	0	0
10112	68401	1	1	1
60426	17573	0	0	0
60426	36960	0	1	0
60459	22656	1	0	0
60459	34257	1	1	1
60750	16694	0	0	0
60750	31642	0	1	0
60340	22481	0	0	0
60340	43377	0	1	0
60760	19982	0	0	0
60760	41667	0	1	0
60708	21284	0	0	0
60708	41524	0	1	0
60481	19202	0	0	0
60481	40708	0	1	0
60447	18470	0	0	0
60447	42183	0	1	0
60889	26135	0	0	0
60889	45289	0	1	0
60311	20079	0	0	0
60311	37009	0	1	0
60204	18607	0	0	0

60204	37353	0	1	0
60830	15668	0	0	0
60830	34493	0	1	0
20232	21344	1	0	0
20232	41062	1	1	1
10140	18813	1	0	0
10140	37389	1	1	1
20188	22955	1	0	0
20188	48889	1	1	1
10210	21578	1	0	0
10210	64653	1	1	1
10069	17057	1	0	0
10069	48873	1	1	1
60225	20466	0	0	0
60225	59156	0	1	0
60352	8465	0	0	0
60352	14828	0	1	0
60465	19862	0	0	0
60465	37982	0	1	0
60351	14652	0	0	0
60351	24324	0	1	0
20194	37979	1	0	0
20194	82766	1	1	1
20152	36887	1	0	0
20152	74154	1	1	1
20001	19550	1	0	0
20001	99982	1	1	1
20043	18509	1	0	0
20043	41892	1	1	1
20236	30666	1	0	0
20236	58835	1	1	1
20229	31724	1	0	0
20229	78161	1	1	1
20041	27299	1	0	0
20041	54367	1	1	1
20246	30688	1	0	0
20246	67250	1	1	1
20063	31759	1	0	0
20063	55714	1	1	1
20245	39059	1	0	0
20245	75690	1	1	1
20044	29182	1	0	0
20044	61538	1	1	1
20286	26063	1	0	0
20286	63158	1	1	1
20158	25561	1	0	0
20158	75568	1	1	1
20035	39492	1	0	0
20035	67544	1	1	1
20116	25302	1	0	0
20116	53614	1	1	1
20213	57959	1	0	0

20213	122047	1	1	1
20214	42201	1	0	0
20214	109573	1	1	1
20192	40325	1	0	0
20192	83928	1	1	1
20215	43314	1	0	0
20215	104651	1	1	1
20179	36428	1	0	0
20179	75573	1	1	1
20185	19130	1	0	0
20185	74482	1	1	1
20119	39967	1	0	0
20119	56304	1	1	1
20039	33523	1	0	0
20039	94112	1	1	1
20033	42880	1	0	0
20033	136410	1	1	1
20151	21859	1	0	0
20151	47384	1	1	1
20173	17282	1	0	0
20173	40789	1	1	1
60707	33772	0	0	0
60707	48106	0	1	0
60271	29913	0	0	0
60271	47297	0	1	0
60875	40605	0	0	0
60875	62804	0	1	0
90177	25323	0	0	0
90177	49980	0	1	0
60705	25696	0	0	0
60705	50286	0	1	0
60880	14490	0	0	0
60880	69444	0	1	0
90186	18245	0	0	0
90186	37361	0	1	0
60847	23227	0	0	0
60847	67835	0	1	0
10153	24545	1	0	0
10153	50667	1	1	1
10162	41096	1	0	0
10162	74450	1	1	1
10201	17896	1	0	0
10201	61037	1	1	1
10202	23053	1	0	0
10202	50779	1	1	1
60817	25150	0	0	0
60817	49138	0	1	0
10108	23517	1	0	0
10108	57600	1	1	1
10194	22026	1	0	0
10194	55599	1	1	1
10018	27614	1	0	0

10018	54815	1	1	1
10129	19268	1	0	0
10129	41725	1	1	1
10234	20523	1	0	0
10234	50557	1	1	1
10040	32127	1	0	0
10040	63116	1	1	1
10137	18913	1	0	0
10137	41935	1	1	1
10104	27306	1	0	0
10104	49001	1	1	1
10163	22049	1	0	0
10163	47165	1	1	1
10024	32330	1	0	0
10024	59859	1	1	1
10029	25635	1	0	0
10029	64894	1	1	1
10228	37523	1	0	0
10228	43211	1	1	1
10080	41701	1	0	0
10080	41667	1	1	1
10081	34744	1	0	0
10081	69185	1	1	1
10124	25793	1	0	0
10124	57994	1	1	1
10020	39372	1	0	0
10020	71002	1	1	1
10041	26374	1	0	0
10041	60372	1	1	1
10117	14569	1	0	0
10117	48483	1	1	1
10190	24609	1	0	0
10190	47814	1	1	1
10034	28969	1	0	0
10034	45120	1	1	1
10116	24651	1	0	0
10116	76941	1	1	1
10039	29778	1	0	0
10039	66452	1	1	1
10126	23957	1	0	0
10126	49927	1	1	1
10037	21915	1	0	0
10037	41096	1	1	1
10131	25386	1	0	0
10131	48094	1	1	1
10182	28956	1	0	0
10182	53711	1	1	1
10002	22350	1	0	0
10002	51485	1	1	1
10216	22133	1	0	0
10216	33377	1	1	1
10142	19230	1	0	0

10142	91343	1	1	1
10032	25165	1	0	0
10032	62699	1	1	1
10219	24625	1	0	0
10219	49499	1	1	1
10103	22210	1	0	0
10103	49332	1	1	1
10139	21834	1	0	0
10139	49579	1	1	1
10174	20874	1	0	0
10174	45247	1	1	1
10144	20408	1	0	0
10144	46617	1	1	1
10012	28856	1	0	0
10012	65854	1	1	1
10220	23954	1	0	0
10220	65371	1	1	1
10033	23961	1	0	0
10033	53386	1	1	1
10005	31077	1	0	0
10005	51232	1	1	1
10003	30274	1	0	0
10003	65996	1	1	1
10118	25693	1	0	0
10118	101956	1	1	1
10027	24236	1	0	0
10027	60633	1	1	1
10025	34398	1	0	0
10025	73913	1	1	1
60730	23406	0	0	0
60730	49241	0	1	0
60993	23576	0	0	0
60993	45652	0	1	0
10023	18094	0	0	0
10023	37308	0	1	0
60555	16352	0	0	0
60555	53279	0	1	0
10110	16739	0	0	0
10110	34216	0	1	0
60451	12593	0	0	0
60451	44929	0	1	0
10247	23503	1	0	0
10247	43250	1	1	1
60456	13494	0	0	0
60456	34841	0	1	0
10017	30783	1	0	0
10017	51636	1	1	1
60192	17290	0	0	0
60192	28893	0	1	0
10114	28409	1	0	0
10114	47414	1	1	1
60460	21331	0	0	0

60460	40399	0	1	0
60428	23541	0	0	0
60428	45384	0	1	0
60885	21697	0	0	0
60885	40452	0	1	0
90003	49740	0	0	0
90003	63349	0	1	0
90009	20648	0	0	0
90009	44381	0	1	0
60776	10524	0	0	0
60776	25963	0	1	0
40042	29315	0	0	0
40042	54550	0	1	0
40060	20498	0	0	0
40060	48076	0	1	0
40043	22097	0	0	0
40043	45861	0	1	0
40041	19282	0	0	0
40041	52589	0	1	0
40058	13935	0	0	0
40058	59109	0	1	0
40074	21155	0	0	0
40074	48451	0	1	0
40065	20378	0	0	0
40065	48693	0	1	0
10043	19831	0	0	0
10043	38167	0	1	0
10047	32863	0	0	0
10047	84935	0	1	0
10049	48017	0	0	0
10049	55814	0	1	0
10238	35436	0	0	0
10238	60824	0	1	0
10046	29572	0	0	0
10046	49224	0	1	0
10207	56250	0	0	0
10207	124060	0	1	0
10232	28291	0	0	0
10232	44996	0	1	0
10054	29190	0	0	0
10054	49227	0	1	0
60395	19493	0	0	0
60395	41383	0	1	0
60394	22109	0	0	0
60394	38719	0	1	0
60096	18355	0	0	0
60096	33486	0	1	0
60578	14586	0	0	0
60578	32941	0	1	0
60382	23382	0	0	0
60382	37361	0	1	0
60407	18497	0	0	0

60407	35798	0	1	0
60766	5210	0	0	0
60766	30668	0	1	0
40034	18947	0	0	0
40034	35597	0	1	0
40033	18115	0	0	0
40033	51212	0	1	0
40021	20468	0	0	0
40021	46209	0	1	0
40051	16815	0	0	0
40051	38465	0	1	0
60652	19365	0	0	0
60652	40493	0	1	0
60966	18453	0	0	0
60966	35404	0	1	0
60539	12289	0	0	0
60539	28085	0	1	0
60274	17849	0	0	0
60274	38106	0	1	0
60783	25370	0	0	0
60783	33636	0	1	0
60952	21434	0	0	0
60952	54071	0	1	0
60780	18884	0	0	0
60780	41446	0	1	0
60521	21434	0	0	0
60521	36405	0	1	0
60518	18264	0	0	0
60518	34879	0	1	0
60120	20090	0	0	0
60120	43367	0	1	0
61004	15483	0	0	0
61004	42428	0	1	0
60010	12817	0	0	0
60010	30079	0	1	0
60785	15559	0	0	0
60785	28962	0	1	0
60784	13029	0	0	0
60784	50223	0	1	0
60606	30976	0	0	0
60606	63927	0	1	0
60160	17669	0	0	0
60160	42978	0	1	0
60779	18678	0	0	0
60779	39468	0	1	0
60921	15300	0	0	0
60921	42294	0	1	0
60644	14930	0	0	0
60644	35962	0	1	0
60157	14334	0	0	0
60157	34221	0	1	0
60636	14717	0	0	0

60636	34210	0	1	0
60794	17650	0	0	0
60794	41358	0	1	0
60587	7444	0	0	0
60587	36559	0	1	0
60588	9917	0	0	0
60588	6429	0	1	0
60516	15072	0	0	0
60516	34868	0	1	0
60023	14142	0	0	0
60023	31429	0	1	0
60512	15204	0	0	0
60512	28846	0	1	0
60513	14971	0	0	0
60513	28229	0	1	0
60635	15364	0	0	0
60635	32276	0	1	0
60056	10668	0	0	0
60056	31132	0	1	0
60047	17178	0	0	0
60047	34015	0	1	0
60068	6757	0	0	0
60068	53092	0	1	0
100154	16734	0	0	0
100154	80000	0	1	0
40047	74589	0	0	0
40047	160000	0	1	0
40038	18837	0	0	0
40038	35912	0	1	0
100156	4862	0	0	0
100156	32417	0	1	0
80033	9791	0	0	0
80033	30045	0	1	0
80005	7329	0	0	0
80005	24466	0	1	0
80120	16145	0	0	0
80120	39324	0	1	0
80227	10199	0	0	0
80227	33226	0	1	0
80224	14159	0	0	0
80224	32078	0	1	0
60940	11114	0	0	0
60940	22960	0	1	0
100022	13716	0	0	0
100022	23974	0	1	0
